# A Novel Automated High-Content Analysis Workflow Capturing Cell Population Dynamics from Induced Pluripotent Stem Cell Live Imaging Data

**DOI:** 10.1177/1087057116652064

**Published:** 2016-06-02

**Authors:** Maximilian Kerz, Amos Folarin, Ruta Meleckyte, Fiona M. Watt, Richard J. Dobson, Davide Danovi

**Affiliations:** 1 Centre for Stem Cells and Regenerative Medicine, King’s College London, Tower Wing, Guy’s Hospital, London, UK; 2 Institute of Psychiatry, Psychology and Neuroscience, King’s College London, London, UK; 3 National Institute for Health Research, Biomedical Research Centre for Mental Health, and Biomedical Research Unit for Dementia at South London and Maudsley NHS Foundation, London, UK; 4 Farr Institute of Health Informatics Research, UCL Institute of Health Informatics, University College London, London, UK

**Keywords:** live imaging, CellProfiler, HipDynamics, iPSC, high-content screening

## Abstract

Most image analysis pipelines rely on multiple channels per image with subcellular reference points for cell segmentation. Single-channel phase-contrast images are often problematic, especially for cells with unfavorable morphology, such as induced pluripotent stem cells (iPSCs). Live imaging poses a further challenge, because of the introduction of the dimension of time. Evaluations cannot be easily integrated with other biological data sets including analysis of endpoint images. Here, we present a workflow that incorporates a novel CellProfiler-based image analysis pipeline enabling segmentation of single-channel images with a robust R-based software solution to reduce the dimension of time to a single data point. These two packages combined allow robust segmentation of iPSCs solely on phase-contrast single-channel images and enable live imaging data to be easily integrated to endpoint data sets while retaining the dynamics of cellular responses. The described workflow facilitates characterization of the response of live-imaged iPSCs to external stimuli and definition of cell line–specific, phenotypic signatures. We present an efficient tool set for automated high-content analysis suitable for cells with challenging morphology. This approach has potentially widespread applications for human pluripotent stem cells and other cell types.

## Introduction

Phase-contrast and confocal microscopy have greatly facilitated the progress of cell biology.^1^ Understanding cellular morphology is especially important in order to unravel subcellular signaling mechanisms.^2^ Modern robotic microscope systems allow scientists to acquire a large number of images relating to several diverse conditions within a short time period. Hence, automated and robust image segmentation, object detection, and the subsequent cellular morphometry are now integral parts of microscopic analyses. Furthermore, because of the introduction of time dimensionality, aggregating biological information extracted from live imaging data and from endpoint high-resolution imaging is a challenging problem with a much sought-after solution. In this article, we present an enhanced workflow combining two methodologies. We report an improved CellProfiler-based image segmentation pipeline, hereafter referred to as image analysis pipeline for single-channel images (IAPSCI), that enables robust identification of induced pluripotent stem cells (iPSCs), cells with highly variable morphology on single-channel, phase-contrast images. In addition, we present an open-source solution, named HipDynamics, to capture cell population changes from live imaging data enabling collation and aggregation to endpoint imaging data sets via dimensionality reduction.

Automated object segmentation of brightfield and phase-contrast, gray-scale cellular images is a well-known challenge, because of the large variability in cell morphology and poor edge-background contrast. While strategies to optimize segmentation exist, basic intensity thresholding is still one of the principal methods employed in object detection pipelines, often complemented by applying edge detection, active masks, Markov random fields, or support vector machines.^3^

CellProfiler (CP) is a cellular image analysis software, developed and generously made open source by the Broad Institute.^4^ Tested image processing, segmentation, and object measurement algorithms are arranged in series (i.e. where one module’s output is the input of the next) forming an image analysis pipeline.^5^ The software enables quick prototyping of pipelines and automated analysis of thousands of images.

CP’s conventional method of cell object segmentation is by employing the IdentifyPrimaryObjects module, which is based on identifying a high-contrast (typically fluorescent) subcellular feature (typically the nucleus) for each cell. As the second step, the IdentifySecondaryObject module extends the former segmented primary objects until an edge is detected.^4^ This methodology is supported by robust and tested algorithms.^6,7^ However, in the case of comparatively low-contrast, gray-scale, single-channel images of cells with highly variable morphology and where subcellular features are difficult to identify, CP’s conventional object segmentation technique proves largely inadequate.

High-content image data sets often contain thousands of images and several orders of magnitude more cells. Furthermore, cell segmentation analysis frameworks often produce dozens, sometimes hundreds, of phenotypic features to quantify any given cell. Thus, in the case of high-content screens, data sets can become unwieldy and lead researchers to ignore many morphological measures that may have revealing patterns in downstream analyses.^8^

A common methodology to account for this problem is to employ per-image (per-field, per-well) data reduction techniques or per-cell classifications. The latter techniques may average cellular measures, or compare cell populations by evaluating corresponding morphology measure distributions, using metrics such as Kuiper and Kolmogorov-Smirnov tests.^9–11^ Per-field (or per-well) techniques usually require predominant cell population changes in order to be captured. Conversely, per-cell classification techniques allow identification of small changes in a given population, albeit with a requirement for supervised or semisupervised learning. This is cell type specific, time-consuming, demands the user’s intervention, and it requires a formulated hypothesis regarding distinct cell states.^12^

Besides the sheer number of feature types and their values, an additional challenge in live imaging high-content analysis is the introduction of a third dimension, time. Depending on the experiment, live imaging may or may not increase the size of raw data produced, yet it almost inevitably increases the complexity of appropriate data extraction when collated with data from endpoint images or other types of datasets (i.e., genomics, gene expression, and/or proteomics). Current tools are unable to reduce time dimensionality, leaving researchers to focus on specific time points, which can result in loss of information.

In this study, we set out to confront these two problems, namely, the robust segmentation of single-channel images and dimension reduction of live imaging data. We present IAPSCI, an improved object segmentation methodology for CP that results in robust cell segmentation on single-channel images. We also introduce HipDynamics, an R-based, open-source project that reduces time-dependent, CP-generated cell morphology measures for enhanced downstream analyses. IAPSCI and HipDynamics are herein combined as a novel, automated, two-step workflow.

## Methods

### Cell Culture, Green Fluorescent Protein Transduction, Image Acquisition

Two human iPSC lines were analyzed. CTR-M2-O5 was a kind gift of Jack Price, and IELY was provided by Tamir Rashid and Ludovic Vallier.^13^ Feeder-free CTR-M2-O5 cells (passage 45) were cultured on Geltrex, and CTR-M2-O5 (passage 46) cells were cultured on vitronectin in E8 medium (Gibco, Waltham, MA). Cultures of both cell lines were rinsed with HBSS (Life Technologies, Carlsbad, CA) and then incubated for 8 min with Accutase (Biolegend, San Diego, CA) to create a single-cell suspension. Cells were then seeded on three different concentrations of fibronectin (FN), as described by Leha et al.^14^ IELY was cultured, dissociated, and seeded on 96-well plates as described by Leha et al.^14^ Cells were transduced with Baculovirus based CellLight Nucleus-GFP, BacMam 2.0 (Thermo Fisher Scientific, Waltham, MA), according to the manufacturer’s instructions, to generate green fluorescent nuclear reference points with low efficiency. Images were acquired hourly for a total of 24 h using an IncuCyte Zoom (Essen Bioscience, Ann Arbor, MI) with a 10× objective and extracted as described by Danovi et al.^15^ After 24 h, the ratio of transduced to untransduced cell lines was comparable between experiments.

The following subsections of the Methods section focus on the image analysis pipelines. They detail the tested conventional CP image analysis or image analysis pipeline for multichannel images (IAPMCI), the parallelized IdentifyPrimaryObjects modules CP image analysis or image analysis pipeline for single-channel images (IAPSCI) and the method used to compare the two.

### IAPMCI: Image Analysis Pipeline for Multichannel Images

The standard IdentifyPrimaryObjects module CP image segmentation methodology IAPMCI was run with the following modules and specifications. Parameters are left at default unless specified.

1. LoadImages. The module loads images one by one within a specified folder and feeds them into the pipeline. Specifications: Input image file location: YourImageSetLocation.
2. UnmixColors. The module creates separate gray-scale images from one color image. Specifications: Red absorbance: 0; Green absorbance: 1; Blue absorbance 0.
3. ImageMath. The module is able to perform mathematical operations, such as addition and subtraction, on the image. Specifications: Operation: Invert.
4. ImageMath. Specifications: Operation: Subtract, Multiply the result by: 2.5.
5. ApplyThreshold. The module removes pixels below or above a particular intensity. Specifications: Select the output image type: Grayscale; Set pixels below or above the threshold to zero: Below; Threshold strategy: Manual; Manual threshold: 0.55.
6. ImageMath. Specifications: Operation: Invert.
7. IdentifyPrimaryObjects. A module that identifies specific features within an image, usually a subcellular entity. Specifications: Typical diameter of objects: 5 to 35, Threshold strategy: Automatic.
8. Apply Threshold applied on entire gray-scale image of step 1. Specifications: Set pixels below or: Below threshold, Threshold strategy: Manual, Manual threshold: 0.33.
9. ImageMath applied on image from step 1. Specification: Multiply the result by: 4.0.
10. IdentifySecondaryObjects. The module finds dividing lines in the surrounding of the location specified by the IdentifyPrimaryObjects module. Specifications: Select method of secondary object identification: Watershed - Gradient, Threshold strategy: Global, Threshold method: Otsu, Threshold correction: 0.4, Lower and upper: 0.0 to 0.7.
11. OverlayOutlines. The module allows for overlay of the outline of an object onto the original input image. Specifications: Overlay secondary objects and output image of step 1.
12. SaveImages. The modules allows saving its input image to a specified location. Specification: Specify an output folder.
13. ExportToSpreadsheet. The module allows exporting metadata and measurements of an object type to a spreadsheet. Specifications: Output file location: Specify output location, Press button to select measurements: Measurements of secondary objects.

The following five modules are solely used for HipDynamics processing and following downstream analyses.

14. MeasureObjectIntensity. The module extracts 17 intensity features for each object identified based on the corresponding gray-scale image. Specifications: Objects: Objects of step 10; Image: output image of step 1.
15. MeasureObjectSizeShape. The module measures 17 area and shape features of objects. Specifications: Objects: Objects of step 10.
16. MeasureObjectNeighbours. The module computes seven features of an object’s neighbors. Specifications: Object: Objects of step 14; Neighboring objects: Objects of step 10.
17. MeasureRadialDistribution. Measures features about the radial distribution intensities of objects. Specifications: Objects: Objects of step 10; Image: output image of step 1.
18. ExportToDatabase. The module exports all measurements to a SQL-compatible database. Specifications: Experiment name: NameToYourLikingX; Database specification (an existing database (DB) needs to be present. Find more information about appropriate DBs below.); Table Prefix: NameToYourLikingY; Which objects should be used of locations: Objects of step 10.

The DB employed for the presented workflow was a MySQL version 5.5 DB (Oracle, Inc.). Before running the presented workflow, a new user with writing permission as well as a new schema has to be created. This process is necessary only once. The schema, its data, and user will be maintained during future runs of the presented workflow. CP automatically exports structured data into the MySQL DB, which is picked up by HipDynamics for further processing. The MySQL’s user details, IP address, and schema name need to be supplied to CP’s ExportToDatabase module and added to hipDynamic.R file to guarantee successful data transferal and persistence.

### IAPSCI: Image Analysis Pipeline for Single-Channel Images

The improved Parallelised IdentifyPrimaryObjects modules CP image segmentation methodology uses conventional CP modules; however, it processes several primaryObjectsIdentification modules each parameterized with incremental length scale ranges in parallel. This is followed by an integration step to prioritize the largest object at a given location and discarding of subobjects. To merge the objects from each IdentifyPrimaryObject module, objects are contracted by one pixel from their boundary and the stack merged. This is to avoid merging distinct neighboring objects and solely focuses on overlapping segmentations. Once merged, the objects are resegmented and expanded by one pixel to the original size, followed by object measurement and result output.

IAPSCI was run with the following modules and specifications. Unless otherwise specified, parameters of each module were left at default:

1. LoadImages. Specifications: Input image file location: location of image test set.
2. ImageMath. Specifications: Operation: Invert. The following series of modules is repeated three times, but with different specifications. When describing the specifications for each module, * denotes the first, ** denotes the second, and *** the third repetition. If no * is given, the same specification was used in all repetitions.
3. ApplyThreshold. Specifications: Select the output image type: Grayscale; Set pixels below or above the threshold to zero: Below; Threshold strategy: Manual; Manual threshold: 0.78*, 0.75**, 0.74***.
4. ImageMath. Specifications: Operation: Invert. All other parameters were left at default.
5. IdentifyPrimaryObjects.. Specifications: Typical diameter of objects: 5 to 20*, 21 to 40**, 41 to 65***; Threshold strategy: Automatic.
6. ReassignObjectNumber. The module unifies unique objects into one provided they are below a given distance away from each other. Specifications: Unification Method: Distance; Maximum Distance: 2 pixels.

The following array of modules is run after the three-cycle repetition.

7. MaskObjects. The module allows removal of objects that are outside or inside a specified region. Specifications: Select objects to be masked: Objects identified in repetition 1; All other parameters were left at default. Mask using a region defined by: Objects; Select masking object: Objects identified in repetition 2.
8. MaskObjects. Specifications: Select objects to be masked: Objects remaining in step 8; Mask using a region defined by: Objects; Select masking object: Objects identified in repetition 3.
9. MaskObjects. Specifications: Select objects to be masked: Objects identified in repetition 2; Mask using a region defined by: Objects; Select masking object: Objects identified in repetition 3.
10. ConvertObjectsToImage. The module transforms all objects identified back into an image format. Specifications: Input objects: Objects remaining after step 9; Color format: Binary.
11. ConvertObjectsToImage. Specifications: Input objects: Objects remaining after step 10; Color format: Binary.
12. ConvertObjectsToImage. Specifications: Input objects: Objects remaining after repetition 3; Color format: Binary.
13. ImageMath. Specifications: Operation: Add; Images: Binary images generated in step 11, 12 and 13. All other parameters were left at default.
14. IdentifyPrimaryObjects. Specifications: Typical Diameter: 1 to 40; Threshold strategy: Binary.
15. OverlayOutlines. Specifications: Overlay objects of step 14 and output image of step 1.
16. SaveImages. Specification: Specify an output folder.
17. ExportToSpreadsheet. Specifications: Output file location: Specify output location, Press button to select measurements: Measurements of secondary objects.

The following five modules are not necessary for cell segmentation procedures and performance evaluation but are solely used for HipDynamics processing and/or following downstream analyses.

18. MeasureObjectIntensity. Specifications: Objects: Objects of step 14; Image: output image of step 1.
19. MeasureObjectSizeShape. Specifications: Objects: Objects identified in step 14.
20. MeasureObjectNeighbours. Specifications: Object: Objects of step 14; Neighbouring objects: Objects of step 14.
21. MeasureRadialDistribution. Specifications: Objects: Objects of step 14; Image: output image of step 1.
22. Export to Database. Specifications: Experiment name: NameToYourLikingX; Database specification (An existing database needs to be present); Table Prefix: NameToYourLikingY; Which objects should be used of locations: Objects of step 14.

### IAPMCI–IAPSCI Performance Comparison

The Image test set, the CP project file containing the pipeline above, and the R-script used to generate the plots to compare the performance of the two pipelines are available in the online supplements. The performance of the two image analysis pipelines was determined for each image by the number of objects identified and by the analysis of their features. As an image test set, we used phase-contrast images with an additional green channel, referring to cells expressing nuclear green fluorescent protein (GFP). The data exported by the SaveToSpreadsheet was used to produce the Key Performance indicators KPI 1 and KPI 2.

### HipDynamics Methodologies

HipDynamics is an open-source project and can be downloaded from Github.^16^ It allows visualization of dynamic changes in cell populations on a plate, aggregating per well or, when several technical replicates are present from the same experiment, per condition. Here, *condition* refers to the concentration of FN present in a specific well. To fulfill these criteria, HipDynamics performs an optional aggregation of metadata information, such as condition levels, by matching plate and well locations with their metadata. Plots are easily generated, depicting population dynamics of morphological features per well or per condition. Furthermore, the user is presented with a summary file containing quantified trends in a time dimensionality–reduced form. The summary file can be passed on for further downstream analyses or used as a final output to assess cell populations. This approach was developed to facilitate parallel analysis of cell measurement data from live imaging and endpoint imaging. The following pseudocode summarizes the core methodologies employed in HipDynamics to generate its outputs.

A. Pseudocode for computing and visualizing iPSC population dynamics of morphological features.

1 | For each *feature* per well per cell line in aggregated data:
2 | *binSize* := compute dynamic bin size based on maximum and minimum feature values in aggregated data
3 | *bins* := create array of n bins with size *binSize*
4 | For each *hour* in *feature*:
5 | *matrix*[*hour*] := compute histogram from the cellular objects at *hour* based on *bins*
6 | *heatmap* := normalise *matrix*
7 | plot *heatmap*

B. Pseudocode for computing time dimensionality–reduced trends.

1 | For each *feature* per well per cell line in aggregated data:
2 | For each *hour* in *feature*:
3 | *array[hour]* := compute IQR, excluding outliers that lie 3 standard deviations from the mean
4 | *linReg* := perform linear regression on *array*
5 | extract *gradient* and *y-intercept* from *linReg* and emit to summary table

## Results

iPSCs are particularly challenging to segment because of their highly variable morphology and their inherent tendency to come together in clumps. Our project framework establishes a complete suite of solutions to enable characterization of a large panel of iPSCs exposed to diverse extracellular conditions. For this purpose, data from single-channel live images and from endpoint images can be integrated for evaluation alongside other data sets, such as genomics, gene expression, and proteomics. To set up an initial simple workflow, cells were plated on three concentrations of FN (1, 5, and 25 µg/mL) as an extracellular matrix suitable for culturing pluripotent stem cells to build a signature of individual iPSC lines.^14^ In this established assay, we first compared the performance of the novel IAPSCI image analysis pipeline to the conventional IAPMCI. For this purpose, we used a small two-channel live-image set of iPSCs (*n* wells = 54, *n* hours = 24, total *n* images = 1296).^13^ In this particular data set, the second image channel contains green fluorescent nuclear live dye emissions, acting as a reference point for IAPMCI, to allow assessment of the image analysis pipeline’s robustness. The performance evaluation for both pipelines is shown in the following sections and compared against a manual count of fluorescent objects.

### Performance of the Novel Image Analysis Pipeline

**Figure 1.**
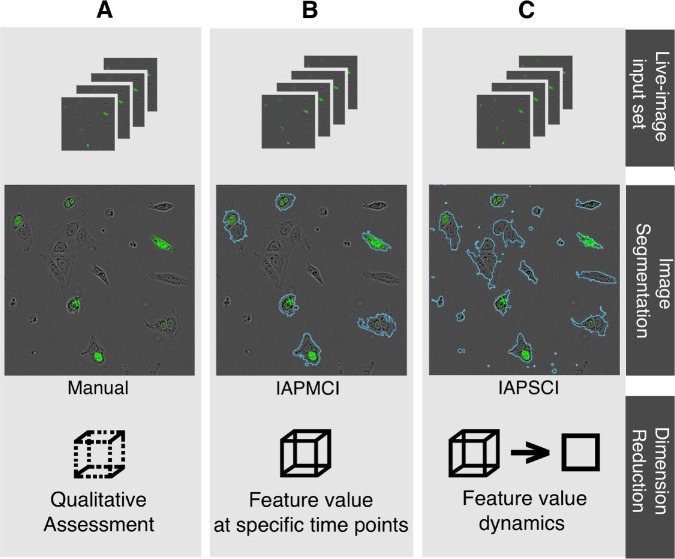
High-level overview of typical live-image analysis workflows compared with the novel one described in this study. (**A**) Manual: Manual cell counting, data curation, and quality assessment. (**B**) Semi-automated workflow with automated image segmentation requiring multichannel images (IAPMCI), followed by manual data curation to account for time dimension. (**C**) Automated workflow: Involves automated image segmentation of single-channel images (IAPSCI), time-dimensionality reduction (HipDynamics), and downstream analyses.

IAPMCI is largely dependent on sufficient reference points to allow reliable edge detection of primary objects within a given cell. Therefore, the efficiency of nuclear staining of iPSCs in the image set dictates its success rate. As a result, iPSCs with a clear fluorescent signal are efficiently detected (**Fig. 1B**). A lack of sufficient reference points can result in inadequate or absent object expansion, leading to high levels of inaccuracy.

Conversely, IAPSCI uses multiple parallelized Identify-PrimaryObject modules to improve the pipeline’s object detection capability (**Fig. 1C**). When employing this solution with incremental iPSC typical diameter ranges, it is possible to detect subcellular features of different sizes. The favored (largest) identified object at a given location is fused with objects in its immediate surroundings to segment one cell into one or as few objects as possible. It is important to note that IAPSCI deliberately fuses cellular clumps into one object.

The same set of images were analyzed by the two pipelines. For segmentation, the conventional pipeline IAPMCI used fluorescent and gray-scale channels, whereas IAPSCI accessed only the gray-scale channel for segmentation. Importantly, IAPSCI was referred to as IAPSCI (GFP) where it was forced to recognize only objects overlapping and/or within the perimeter of the fluorescent green cells, identified by IAPMCI.

**Figure 2.**
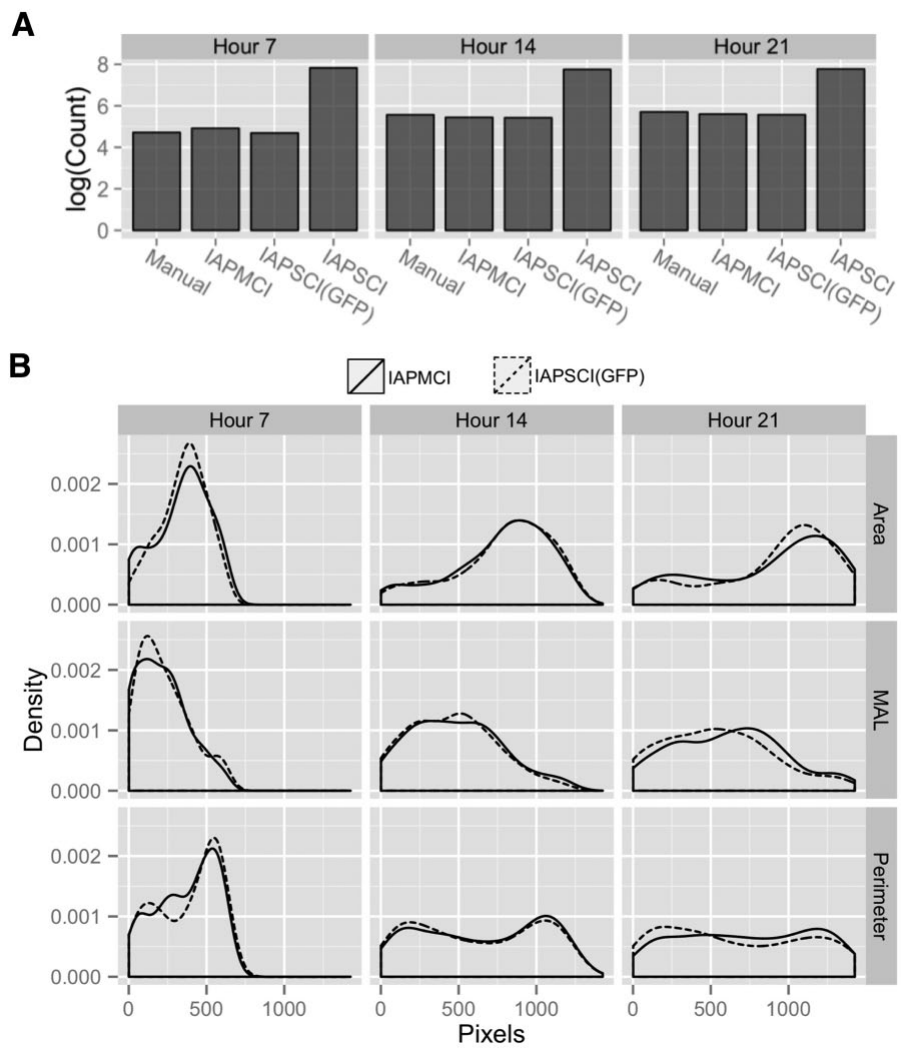
Key performance indicators (KPIs) for quantitative comparison of image analysis pipeline for multichannel images (IAPMCI) and image analysis pipeline for single-channel images (IAPSCI). (**A**) KPI 1: Number of objects identified by manual count, IAPMCI, IAPSCI (green fluorescents protein [GFP]), and IAPSCI at hour 7, 14, and 21 on a log scale. IAPSCI does not depend on expression of GFP and identifies a larger number of objects compared with the others, both similar to a manual count. (**B**) KPI 2: Area, minor axis length (MAL), and perimeter density of all objects identified by IAPMCI and equivalent objects identified by IAPSCI (GFP) at hour 7, 14, and 21. There is an overall similarity in the density for both pipelines demonstrating comparable performance.

First we set to define the accuracy and reliability of the two pipelines in terms of number of objects segmented compared with a manual fluorescent count acting as “ground truth.” **Figure 2A** visualizes the number of objects at 7, 14, and 21 h, identified by IAPMCI, IAPSCI (GFP), and IAPSCI. The manual count used as gold standard refers to the number of objects expressing nuclear GFP, whether within a single cell or in one or several cells adhering to form a clump. The detection accuracy of IAPSCI (GFP) and IAPMCI appears to be similar, due to a near equal number of identified cells for both measures. Importantly, when IAPSCI is run without constraints on GFP-expressing cells, several orders of magnitude more cells are identified.

Next, we wanted to explore the performance of the two pipelines in terms of specific morphological features of the objects segmented. **Figure 2B** shows object area, minor axis length, and perimeter as examples of features from objects segmented by IAPMCI and IAPSCI (GFP). For these features, a shift in distribution toward an increased size is observed with experiment progression (hour 7, 14, and 21), consistent with spreading and clumping of cells over time. A high degree of similarity of these distributions can be observed over 21 h of live imaging. Altogether, these results show that despite the absence of nuclear GFP as a reference, IAPSCI is capable of segmenting cells with challenging morphology.

### Time Dimensionality Reduction of Live-Image Data

**Figure 3.**
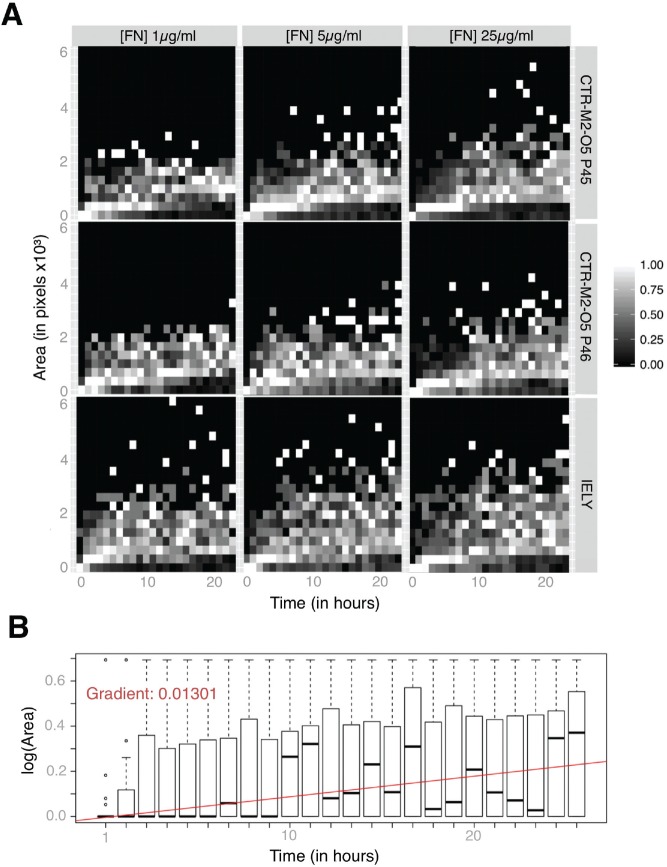
A descriptive example of visualization and dimensionality reduction for distinct features obtained by HipDynamics. (**A**) A condition-level view of the effect of fibronectin (FN) concentrations on area for induced pluripotent stem cell (iPSC) cultures (from top to bottom: CTR-M2-O2 P45, CTR-M2-O2 P45, and IELY). The increase in objects area with time due to spreading and formation of clumps takes place at different rates, depending on the extracellular condition (from left to right: 1, 5, or 25 µg/mL). (**B**) Computation of time dimensionality–reduced data point, the gradient of the linear curve, of the IELY cell line at (FN) 25 µg/mL. The gradient of the red curve is computed using the mean of all interquartile ranges (IQRs) for each hour. The same methodology is applied at every condition level for each cell line to all features.

Data from our chosen pipeline IAPSCI from iPSC over three FN conditions can be obtained from single-channel images, and each feature can be quantified over time. We next set out to explore dimensionality reduction to enable visualization of normalized trends. This is an important step to simplify feature selection, evaluation, and integration of data with other data sets. Based on the observation that on the three FN concentrations, cells appeared to spread/clump in different ways, giving rise to objects with increasing size at different time points after plating, we explored the trends for specific features over time. **Figure 3A** shows an example of HipDynamic’s visualization at the FN condition level for one specific feature (segmented object area). With increasing (FN) concentration, it is possible to observe an accelerated rate of cell area expansion on both tested iPSC lines.

Next we set out to apply dimensionality reduction, opting for a linear regression (LR) applied to each feature, as opposed to retaining all the time points (*n* hours = 24 in our case). The LR’s gradient now accounts for the information on time-related variability (**Fig. 3B**).

**Figure 4.**
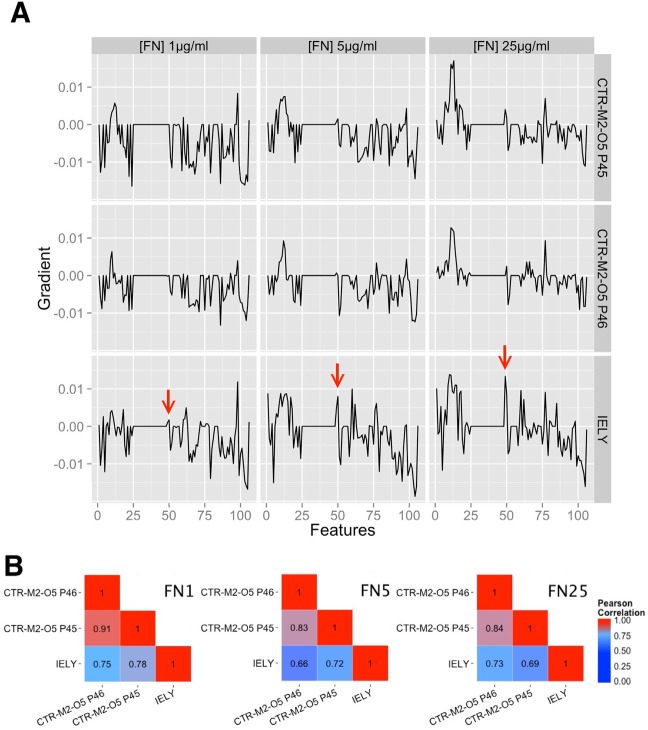
Characterization of cell lines using HipDynamic’s feature signature vectors. (**A**) Cell lines’ signature vectors constructed from the gradients of each measure at different fibronectin (FN) concentrations (from left to right: 1 µg/mL, 5 µg/mL, and 25 µg/mL). Some features are FN dependent, and the IELY signature appears distinct. The red arrows highlight one specific feature (cell edge intensity) as an example. (**B**) The feature signature vectors were assembled in correlation matrices according to their FN concentration (condition level).

While important information can be derived from specific features, such as area over time, we aimed to address whether a cell population’s behavior over time could be quantified using the full breadth of its time-reduced features (*n* = 106) assembled into a gradient signature vector. The signature vectors of the iPSC lines that were used in the image set on IAPSCI at all three condition levels are displayed in **Figure 4A**. Some features appeared to be characteristic of one cell culture and/or (FN) concentration. We next set out to assess whether the signature vector was able to quantify the similarity between cell lines by computing correlation matrices of the cell cultures for each condition level (**Fig. 4B**). All correlations show a positive similarity in the range of 0.66 and 0.91, where a higher correlation suggests a higher similarity in feature signature vector and hence cell line characteristic.

Importantly, the results of **Figure 4A** and **B** show that independent equivalent cultures of the same cell line (CTR-M2-O5 P45 and P46) are more strongly correlated compared with IELY, a nonrelated cell line. In fact, in each feature condition, IELY is distinct from CTR-M2-O5 (**Fig. 4B**). A list of all 106 features as well as a table with all feature vectors can be found in the **Supplementary Material**. Altogether, these results indicate that cell line–specific signatures can be captured, observed, quantified, and compared with live images using the methodology we have developed.

## Discussion

The cell line–specific dynamic signatures generated via HipDynamics can now be applied to large panels of iPSCs. The cells show dramatic changes over time, from simple round objects at the time of seeding stage to complex, irregular, and highly spread morphologies at later time points.

IAPSCI is a novel and unconventional approach to detect objects in a robust manner, making use of existing CP modules. The pipeline’s detection accuracy is solely dependent on image quality and is a useful tool for fast, automated, non–machine learning, high-content, live and/or endpoint imaging experiments in presence of challenging cell morphologies. We conclude that IAPSCI has a similar accuracy and robustness in detecting objects on single-channel, phase-contrast, cellular images to IAPMCI, which in turn is dependent on a minimum of two channels.

IAPSCI and conventional pipelines require input parameters for optimal segmentation. Manual pipeline optimization is necessary, due to microscope- and cell type–specific exposure settings that generate images with different brightness and focus. The number of input parameters for IAPSCI was kept to a minimum (*n* = 3 in step 3) for accelerated image analysis pipeline optimization. In direct comparison, IAPSCI requires fewer parameters than IAPMCI.

The low transduction rate resulted in poor efficiency of GFP expression, especially during the first hours of imaging. IAPMCI tends to oversegment large parts of the image in the case of a suboptimal number of fluorescent reference points during early hours after plating. We chose hour 7 as our first evaluation point to minimize this effect. The performance comparison shows that IAPSCI is as accurate as IAPMCI, with the significant advantage of not being dependent on fluorescent live dyes. This is particularly relevant in experiments in which the application of live dyes may affect cell behavior, introduce bias through a priori feature selection, and exert financial strain due to live-dye costs.

Importantly, IAPSCI cannot detect cells within clumps. The reason lies in the similarity of image intensity for adjacent cells. Instead, IAPSCI detects a clump as one object. Nevertheless, for iPSCs, the properties of cell adhesion and clump morphology are very useful signatures that appear to be cell line specific.

Finally, we report here the unique advantage offered by HipDynamics for researchers to view their segmented and measured data sets. Multiple view level visualization allows identification of feature-based trends, relationships, and anomalies that may have been easily missed with current methodologies. This approach offers a valid alternative to computationally expensive machine learning methods, such as PhenoDissm, a software package using support vector machines to deduce phenotypic dissimilarity of cells in a high-content, image-based environment.^17^ In addition, easy comparison with endpoint imaging data, mitigation of feature selection, and application of downstream analyses are enabled by HipDynamics’ time dimensionality reduction capabilities.

HipDynamics uses LR to reduce multiple time-dependent data points into one. The feature vectors shown in **Figure 4A** suggest that LR is sufficient to detect cell line– and condition level–specific changes. LR also produces an intercept measure that in our preliminary observations appears largely independent of FN concentration and cell line (not shown). Further work would be required to explore the relevance of this value. Rather than applying LR, it could also be possible to employ a logistic regression with multiple polynominals. While the latter may result in a more sensitive feature vector, it also bears the danger of overfitting to one specific cell culture, thus compromising the potential of computing a generalizable feature signature vector for specific cell lines. As a result, we chose to employ LR in the present studies.

As future work, we aim to develop a standalone CellProfiler module that incorporates IAPSCI and its refined methodology. Furthermore, we are packaging it together with HipDynamics into a scalable web application that can be used intuitively and help to reduce the burden of high-content data management.

The validated platform described in this study is likely to further extend its value to other cell types, including cells with different morphologies, and readily offers unique capabilities to characterize large panels of human pluripotent stem cells.

## Supplementary Material

Supplementary material

## Supplementary Material

Supplementary material

## Supplementary Material

Supplementary material

## Supplementary Material

Supplementary material

## Supplementary Material

Supplementary material

## Supplementary Material

Supplementary material

## Supplementary Material

Supplementary material

## Supplementary Material

Supplementary material

## Supplementary Material

Supplementary material

## Supplementary Material

Supplementary material

## Supplementary Material

Supplementary material
